# Pericardial Patch Angioplasty Heals via an Ephrin-B2 and CD34 Positive Cell Mediated Mechanism

**DOI:** 10.1371/journal.pone.0038844

**Published:** 2012-06-13

**Authors:** Xin Li, Caroline Jadlowiec, Yuanyuan Guo, Clinton D. Protack, Kenneth R. Ziegler, Wei Lv, Chenzi Yang, Chang Shu, Alan Dardik

**Affiliations:** 1 Department of Surgery and the Interdepartmental Program in Vascular Biology & Therapeutics, Yale University School of Medicine, New Haven, Connecticut, United States of America; 2 Department of Vascular Surgery, Xiangya Second Hospital of Central South University, Changsha, Hunan, China; 3 Department of Vascular Surgery, Shandong University School of Medicine, Jinan, Shandong, China; 4 Veterans Affairs Connecticut Healthcare Systems, West Haven, Connecticut, United States of America; University of Padova, Italy

## Abstract

**Objective:**

Pericardial patches are commonly used in vascular surgery to close arteriotomies. The mechanism of early healing after patch implantation is still not well defined. We used a rat aortic patch model to assess pericardial patch healing and examined Ephrin-B2, a marker of arterial identity, expression within the post-implantation patch. We also determined whether endothelial progenitor cells (EPC) are associated with early patch healing in the arterial environment.

**Methods:**

Wistar rats (200–250 grams) underwent infrarenal aortic arteriotomy and then closure via bovine or porcine pericardial patch angioplasty. Control groups included subcutaneously implanted patches. Patches were harvested at 0–30 days and analyzed by histology, immunohistochemistry, immunofluorescence and Western blot as well as quantitative PCR.

**Results:**

Prior to implantation, pericardial patches are largely composed of collagen and are acellular. Following arterial implantation, increasing numbers of CD68-positive cells as well as Ephrin-B2 and CD34 dual-positive cells are found within both bovine and porcine pericardial patches, whereas the infiltrating cells are negative for vWF and α-actin. Porcine patches have a luminal monolayer of cells at day 7, compared to bovine patches that have fewer luminal cells. Subcutaneously implanted patches do not attract Ephrin-B2/CD34-positive cells. By day 30, both bovine and porcine pericardial patches develop a neointima that contains Ephrin-B2, CD34, and VEGFR2-positive cells.

**Conclusion:**

Both CD68-positive and Ephrin-B2 and CD34 dual-positive cells infiltrate the pericardial patch early after implantation. Arteriotomy closure via pericardial patch angioplasty shows patch adaptation to the arterial environment that may involve a foreign body response as well as localization of EPC. Arterial remodeling of pericardial patches support endothelialization and may represent a paradigm of healing of scaffolds used for tissue engineering.

## Introduction

The use of patch angioplasty during carotid endarterectomy and other arterial closure has become standard in vascular surgery as patch use is associated with improved patient outcomes including reduced risk of restenosis [1], [2], [3]. Debates regarding the optimal patch material, however, continue, and have prompted investigation into which material offers optimal performance. The bovine pericardial patch was first introduced in the 1990s and has become a commonly used material for closure of the surgical angioplasty site [4], [5]. Pericardium possesses many merits including biocompatibility, easy handling, minimal suture line bleeding, and ability to perform Doppler investigations through the patch. Interestingly, despite use of bovine pericardial patches in human patients for almost two decades, long-term results for this biomaterial remain poorly documented, and the mechanism of healing after patch implantation likewise remains unknown. In particular, it is not even known whether cells infiltrate the patch or whether these patches support endothelialization when placed into the arterial circulation.

Historically, bioprosthetic heart valves were one of the first successful applications of engineered tissue, and the introduction of gluteraldehyde preservation in 1969 only served to further enhance their clinical utility. Preliminary results using early unfixed bioprosthetic tissues found them to be immunogenic and susceptible to early degradation, weakening, and failure. The application of gluteraldehyde cross-linking to these engineered tissues decreased the observed immunogenicity and likewise increased product durability *in vivo*. The success of this application quickly became evident in the increasingly common usage of bioprosthetic cardiac valves. Similarly, the gluteraldehyde-fixed umbilical cord was also an important successful arterial conduit for human patients with peripheral vascular disease [6], [7].

Along with increased clinical usage came the early observation that high concentrations of gluteraldehyde used for preservation allowed for less *in vivo* remodeling and had an increased tendency to calcify [8]. Newer generation bioprosthetics were able to successfully use lower concentrations of gluteraldehyde while preserving reduced immunogenicity [9]. Modern bioprosthetics, such as the latest generation of the bovine pericardial patch, continue to use low concentrations of gluteraldehyde in combination with decellularization, the removal of cellular and nuclear material; in addition the gluteraldehyde is removed prior to surgical implantation into patients. These methods have led to the most recent generation of bioprosthetic patches that may allow for *in vivo* remodeling and adaptation. Studies using the rat subcutaneous implantation model have found that these properties allow for fibroblast ingrowth and cellular incorporation coupled with preservation of durability [9]. However, it is not known to what extent remodeling and arterial adaptation, such as endothelialization, occur with patch closure, as well as any differences that might occur in pericardial patches that are not fixed with gluteraldehyde.

Prior work in vein grafts has shown the complexity of venous adaptation to the arterial environment [10]. We have previously shown that arterialized vein grafts show increased cellularity and matrix in all layers of the vessel wall, as well as diminished expression of the venous marker Eph-B4, yet they fail to express Ephrin-B2, a marker of arterial identity [11]. Since pericardium is not derived from a vessel, and therefore does not express either Ephrin-B2 nor Eph-B4, it is not clear whether this biomaterial is capable of adopting an arterial identity and expressing Ephrin-B2 [12].

Accordingly, we examined commercially-available pericardial patches in an arterial patch closure model to determine the mechanisms by which these patches support arterial healing. We hypothesize that once placed into an arterial environment, the pericardial patches will support cellular influx and these entering cells express the arterial marker Ephrin-B2 in addition to CD34, a marker of neovascularization; in addition, we hypothesize that pericardial patches support endothelialization of the patch on its luminal surface. We also tested the hypothesis that pericardial patches that are not crosslinked with gluteraldehyde have similar patterns of healing.

## Methods

### Animal Model

**Figure 1:**
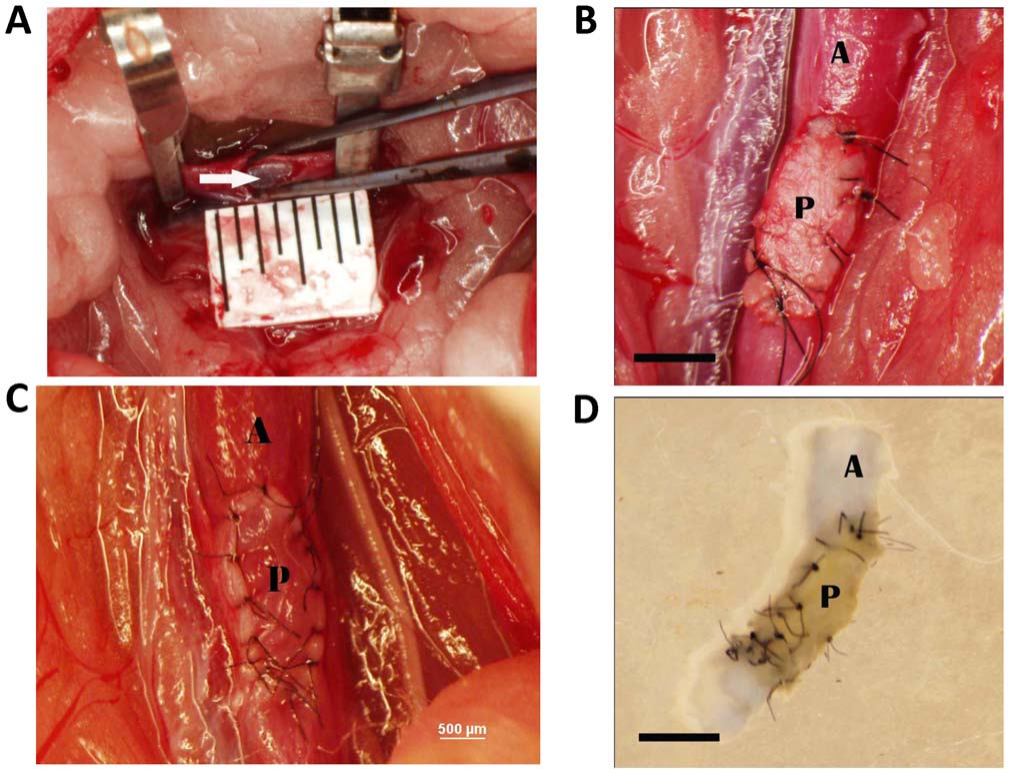
Photomicrographs of the implanted pericardial patch. **A**) The rat aorta after arteriotomy and prior to patch placement; the white arrow shows the glistening intima on the posterior wall; ruler markings are 1mm. Scale bar, 1000 microns. **B**) Completed bovine patch closure. Scale bar, 1000 microns. **C**) Completed porcine patch closure. Scale bar, 500 microns. **D**) Explanted aortic segment containing patch after harvesting and flushing. Scale bar, 1000 microns. In each graph, **A** = aorta; **P** = pericardial patch.

All experiments were approved by the Institutional Animal Care and Use Committee at the Yale University School of Medicine (IACUC# 2010–10896). Young (6–8 week-old) male Wistar rats (mean weight, 209±34 g) were use for patch implantation (n = 24). Anesthesia was performed via isoflurane inhalation with surgery performed using a dissecting microscope (Leica MZ 95, Germany). An abdominal midline incision was made and the infrarenal abdominal aorta was exposed and dissected free of surrounding structures. Following subcutaneous heparin injection (100 U/100 g) the infrarenal aorta was clamped. A longitudinal 3 mm arteriotomy was then made on the anterior aortic wall and a pericardial patch (3 mm×1 mm) was sutured in place using interrupted 10-0 nylon (**Figure** **1**; bovine: Synovis Life Technologies, St. Paul, MN, USA; porcine: Tissue Regenix, York, United Kingdom). After completion of the angioplasty, the micro-clamps were removed and aortic flow was restored. The abdomen was then closed using 5-0 Dacron sutures. Additional age-matched Wistar rats were used for subcutaneous patch implantation (n = 12). Animals were sacrificed on postoperative days 1, 3, 7, or 30. No immunosuppressive agents were given at any time.

### Histology

Rats were anesthetized with isoflurane, and tissues were fixed by transcardial perfusion of phosphate buffered saline (PBS) followed by 10 percent formalin. Tissue was removed and fixed overnight in 10 percent formalin followed by a 24-hour immersion in 70 percent alcohol. Tissue was then embedded in paraffin and sectioned. H&E stained sections were used for cell counts; 4 random representative high power fields were counted from each patch and the mean number of cells in each high power field recorded.

### Immunohistochemistry

Tissue sections were de-paraffinized using xylene and a graded series of alcohols. Sections were then incubated in peroxidase blocking solution and stained using antibodies against von Willebrand Factor (Dako, A0082), α-actin (Thermo Scientific, MS-113), or CD68 (Thermo Scientific, MS-397) overnight at 4°C. Immunohistochemical detection was performed using DAB as well as NovaRED substrate (Vector). Sections were counterstained with Mayer's Hematoxylin.

### Immunofluorescence

Tissue sections were de-paraffinized and stained using primary antibodies against CD31 (BD Pharmingen, 550300), CD34 (R&D, AF4117) Ephrin-B2 (Novus, NBP1-48610), or ED-1 (Abcam). Appropriate fluorophore-conjugated secondary antibodies were selected. An antifade reagent with DAPI nuclear stain (Invitrogen, P-36931) was applied prior to cover slip placement.

### PCR Analysis

Total RNA was isolated from tissue and patches using TRIzol Reagent (Invitrogen) and RNA was cleaned using the RNeasy Mini kit with digested DNase I (QIAGEN). For quantification of total RNA, each sample was measured using the RiboGreen RNA Assay kit (Invitrogen). RT was performed using the SuperScript III First-Strand Synthesis Supermix (Invitrogen) according to the manufacturer's instructions. Real-time quantitative PCR was performed using SYBR Green Supermix (Bio-Rad Laboratories) and amplified for 40 cycles using the iQ5 Real-Time PCR Detection system (Bio-Rad Laboratories). All samples were normalized by GAPDH amplification. The sequences for the primers to amplify Ephrin-B2 and GAPDH were synthesized (Keck Oligonucleotide Synthesis Facility, Yale University, New Haven, CT) and are shown in **Table** **1**. Primers for CD34 were purchased (SABiosciences, PPR55773A-200). Patches were analyzed individually, without combination of samples.

**Table 1:** Primer pairs used in the study.

Name	Forward primer (5′-3′)	Reverse primer (5′-3′)
**Ephrin-B2**	CTGTGCCAGACCAGACCA AGA	CAGCAGAACTTGCATCTTGTC
**GAPDH**	ACAACTTCAGTGTCCTCTGT	TTCGAGTAAAGGACCATACT

### Western Blot Analysis

For protein extraction, the inferior vena cava, the pericardial patch, and the thoracic aorta were carefully harvested separately and snap-frozen in liquid nitrogen. Samples were crushed and mixed with buffer including protease inhibitors (Roche, Complete Mini 12108700) prior to sonication (5 sec) and centrifugation (800 g, 20 min). Equal amounts of protein from each experimental group were loaded for SDS-PAGE, followed by Western blot analysis. The membranes were probed with antibodies against Ephrin-B2 (Novus, NBP1-48610), CD34 (R&D, AF4117), or GAPDH (Cell Signaling, 14C10). Membrane signals were detected using the ECL detection reagent (Amersham, Buckinghamshire, UK). Patches were analyzed individually, without combination of samples.

## Results

### Bovine patches

**Figure 2:**
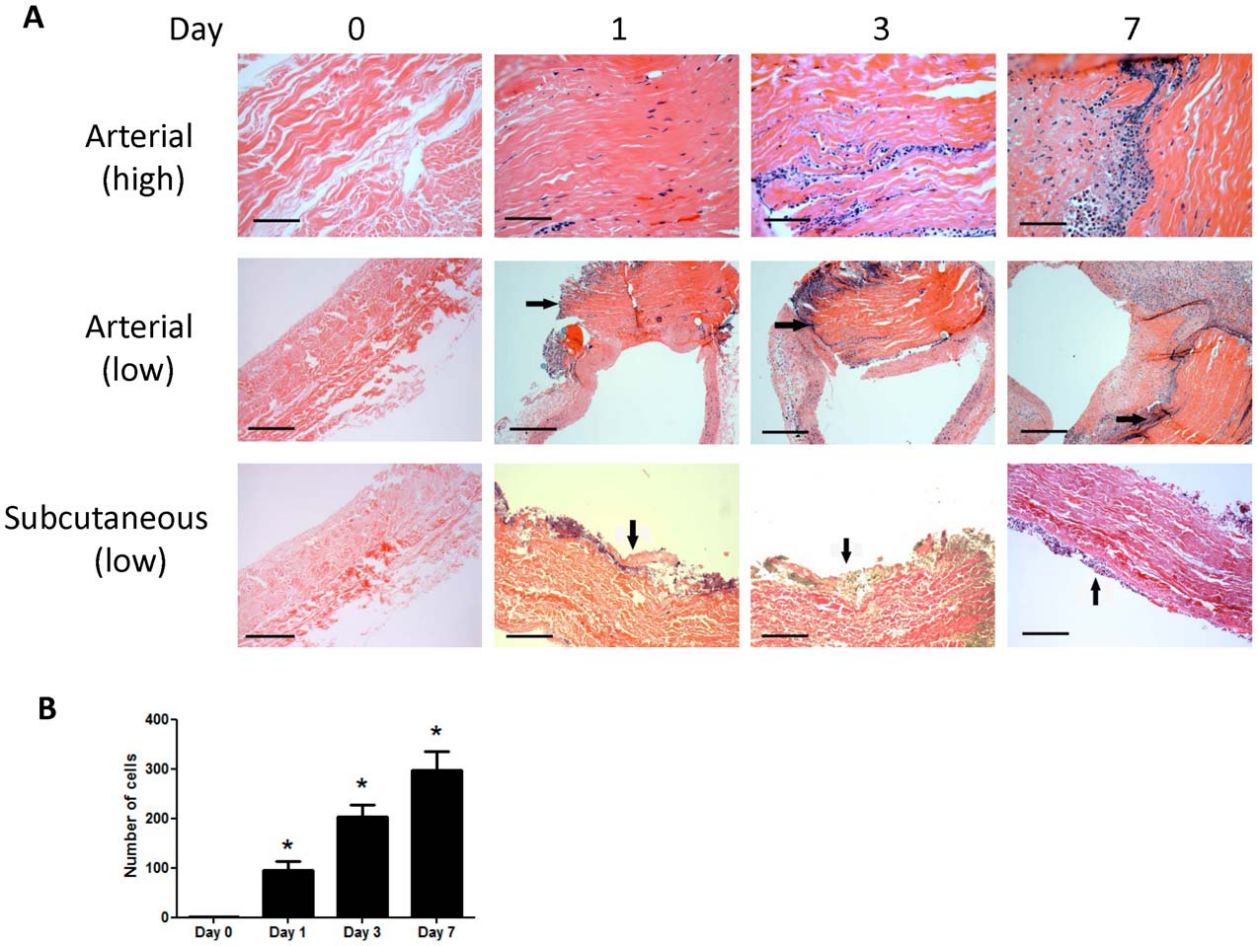
Increasing numbers of infiltrating cells into the arterial patch. **A**) Photomicrographs of bovine pericardial patches, implanted into either an arterial or subcutaneous position, and shown at either high or low magnification. Scale bar, 40 microns. **B**) Graph of cell count in the patch over time; mean of 4 high power fields per patch; n = 2. *, p<0.0001.

To define the pattern of arterial healing with a common clinically-used pericardial patch, bovine pericardial patches were examined in a rat angioplasty model (Vascuguard; Synovis Life Technologies, St. Paul, MN, USA). Prior to implantation bovine pericardial patches are acellular (**Figure** **2**). After implantation into the rat aorta, cells increasingly infiltrated into the patch, between the collagen fibers, with the number of infiltrating cells directly correlating with length of time implanted (**Figure** **2**). Cells were present throughout the angioplasty patch; however, with increasing time, cells migrated more centrally into the interior of the patch compared to the fewer number of cells present on the patch surface (**Figure** **2**). Although subcutaneously implanted patches also had an increasing cellular infiltrate, this infiltrate remained predominantly on the periphery of the patch (**Figure** **2**).

**Figure 3:**
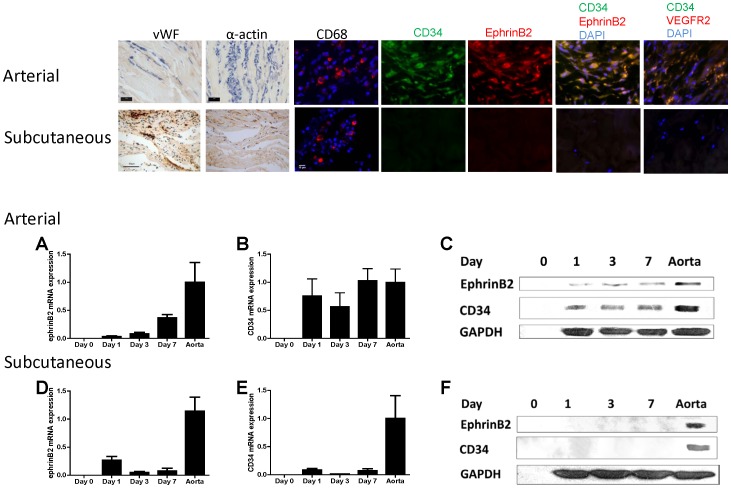
Cells infiltrating into arterial, but not subcutaneous, patches have markers for either macrophages or arterial progenitor cells. Scale bar, 10 microns, except 40 microns for subcutaneous histology. n = 3; representative pictures are shown. Immunofluorescence shows CD34 and Ephrin-B2-positive cells colocalize in the middle of the bovine arterial patch, day 7. Photomicrographs of immunofluorescence: blue color, DAPI; green color, CD34; red color, Ephrin-B2; merge, green + red. n = 3; representative pictures are shown. Scale bar, 10 microns. Similarly, merge of CD34 and VEGFR2 is shown. Bar graphs show increased expression of Ephrin-B2 and CD34 mRNA and protein in arterial, but not subcutaneous, patches. n = 4−5. Panels A, B, D, and E, mRNA; Panels C and F, protein.

Arterial patches were also examined during the first week after implantation for potential markers of cellular identity on the infiltrating cells (**Figure** **3**). At these early time points, an endothelial cell layer was not readily apparent; infiltrating cells were negative for vWF and α-actin, markers of vascular endothelial cells and smooth muscle cells respectively, at all time points (**Figure** **3**). Immunofluorescence for CD31, an alternative vascular endothelial cell marker, confirmed lack of endothelial cells in arterial patches on days 1, 3, and 7 (data not shown). Some of the infiltrating cells stained positively for CD68, consistent with their being macrophages (**Figure** **3**). In comparison, the cells infiltrating into subcutaneously implanted patches developed positive staining for vWF, α-actin and CD68 by postoperative day 7 (**Figure** **3**).

Since some of the cells infiltrating into arterial patches were not positively stained with CD68, we examined these cells for markers that would suggest endothelial or arterial progenitor cell identity. Arterial patches (day 7) were examined using immunofluorescence for both CD34 and Ephrin-B2 (**Figure** **3**). Approximately half of the infiltrating cells were positive for CD34 and the number of cells appeared to increase, in a centrally concentrated position, over time; similarly, Ephrin-B2 was detected on these cells in a mostly similar pattern and that typically colocalized with CD34 (**Figure** **3**). However, cells infiltrating into the subcutaneously implanted patches stained negative for both CD34 and Ephrin-B2 (**Figure** **3**). Similarly, some of the cells infiltrating into arterial patches were dually-positive for CD34 and VEGFR2 by day 7, and none of these cells were detected in subcutaneously implanted patches (**Figure** **3**).

To confirm the presence of Ephrin-B2/CD34 dual-positive cells infiltrating into the arterial patches but not into the subcutaneously placed patches, patches were explanted postoperatively and mRNA extracted from them. qPCR analysis showed that arterial patches had significant expression of both Ephrin-B2 (**Figure** **3A**) and CD34 (**Figure** **3B**) postoperatively, whereas there was minimal Ephrin-B2 (**Figure** **3D**) and CD34 (**Figure** **3E**) expression present in subcutaneously placed patches,. The increase in Ephrin-B2 expression in arterial patches also increased over time when compared to the amount present in the inferior vena cava, although it did not reach the amount present in aorta (data not shown).

Patches were similarly examined for protein expression (**Figure** **3**). Western blot analysis confirmed the presence of Ephrin-B2 and CD34 protein expression within the patches when placed in the arterial (**Figure** **3C**) but not the subcutaneous position (**Figure** **3F**). All levels of protein expression were lower in the angioplasty patch groups when compared to native aorta; no protein expression was found in the pre-implantation patch (**Figures** **3C, 3F**).

### Porcine patches

**Figure 4:**
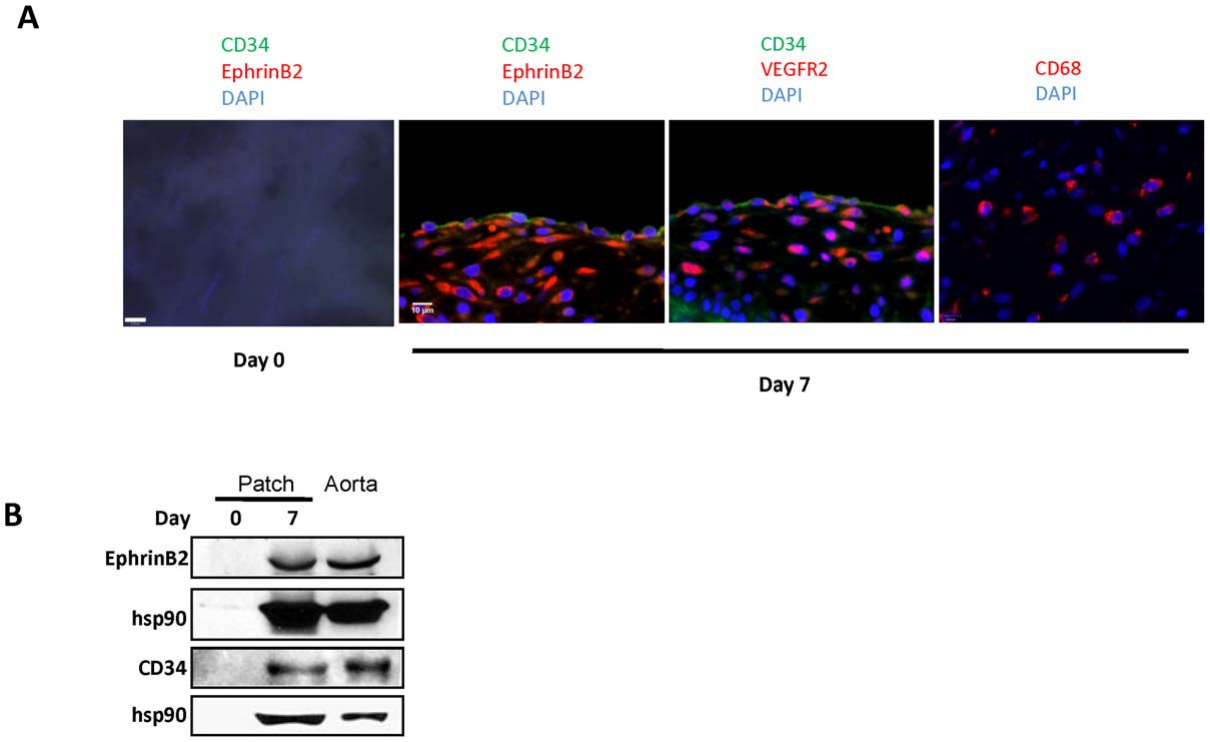
Porcine pericardial patches, day **7.** **A**) Porcine pericardial patches show infiltrating cells positive for CD34, Ephrin-B2, VEGFR2, or CD68; CD34-positive cells are present as a surface monolayer, with some cells dually positive for CD34 and Ephrin-B2 or CD34 and VEGFR2; first panel is the day 0 (preimplantation) control. Photomicrographs of immunofluorescence: blue color, DAPI; green color, CD34; red color, Ephrin-B2 or VEGFR2; merge, green + red. n = 3; representative pictures are shown. Scale bar, 10 microns. **B**) Western blot of Ephrin-B2 and CD34, in porcine pericardial patch, day 0 (preimplantation) or day 7.

To determine the effect of gluteraldehyde cross-linkage of the patch, we examined porcine pericardial patches that are decellularized by a different manufacturing process that does not use gluteraldehyde (dCELL; Tissue Regenix, York, United Kingdom). Examination of these porcine patches with immunofluorescence shows that these patches are acellular prior to implantation (**Figure** **4**), similar to bovine patches (**Figure** **3**). Also similar to bovine patches, cells infiltrating into the porcine patches stained positively for CD34, Ephrin-B2, VEGFR2, or CD68 (**Figure** **4**). However, unlike bovine patches that showed predominantly cellular infiltration into the middle of the patch by day 7 (**Figures 2** **,** **3**), the luminal surface of the porcine patch is covered by cells as early as day 7 after implantation; in addition, these luminal cells are CD34 positive, with some of the cells also being CD34 and Ephrin-B2 dual positive (**Figure** **4**) as well as CD34 and VEGFR2 dual positive (**Figure** **4**). Western blot confirmed that the patches contain Ephrin-B2 and CD34 protein at day 7 (**Figure** **4B**).

**Figure 5:**
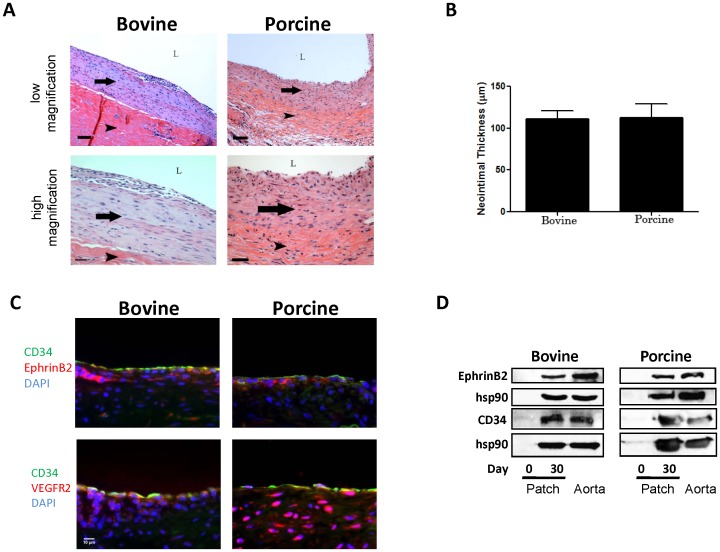
Bovine and porcine pericardial patches, day **30. A**) Representative photomicrographs of bovine and porcine patches, low (10x) and high (40x) magnification, scale bar 80 and 30 (porcine, 40) microns. Arrow shows the neointima; arrowhead shows the patch; L, lumen. **B**) Bar graph of mean neointimal thickness, bovine and porcine patches, day 30. **C**) Photomicrographs of immunofluorescence: blue color, DAPI; green color, CD34; red color, Ephrin-B2 or VEGFR2; merge, green + red. n = 2; representative pictures are shown. Scale bar, 10 microns. **D**) Western blot of Ephrin-B2 or CD34, in bovine or porcine pericardial patch, day 0 (preimplantation) or day 30; n = 2.

Patches were followed to 30 days; both bovine and porcine patches demonstrated a thick layer of luminal neointima (**Figure** **5A**) that was of similar thickness between bovine and porcine patches (**Figure 5B**). Examination of the explanted 30-day patches with immunofluorescence showed that the neointima of both patches was composed of cells that were positive for CD34, Ephrin-B2, and VEGFR2, with minimal differences between bovine and porcine neointima (**Figure** **5C**). Western blot confirmed the presence of Ephrin-B2 and CD34 protein in both bovine and porcine pericardial patches (**Figure** **5D**).

## Discussion

We show that pericardial patches support cell infiltration after implantation into the arterial circulation. Within the first week of implantation of a bovine pericardial patch into an arterial position but not in a subcutaneous position, infiltrating cells express CD68, Ephrin-B2 as well as CD34, but not vWF or alpha-actin, in the middle of the patch and with fewer cells on the luminal surface. Porcine pericardial patches show a similar pattern of cells infiltrating into the middle of the patch, with additional detection of luminal cells that are positive for CD34, Ephrin-B2, and VEGFR2. These results suggest that surgical closure of an artery with a pericardial patch supports arterial remodeling by endothelialization of the luminal surface, at least in the short term, not by ingrowth of endothelial cells or smooth muscle cells, but rather by influx of arterial progenitor cells. However, by 30 days, both bovine and porcine patches develop similar amounts of luminal neointima, suggesting that long term patency is maintained by endothelialization, regardless of patch origin.

### Acquisition of an arterial identity in the patch

A critical finding of this study is the detection of infiltrating cells that express the arterial identity molecule, Ephrin-B2, as well as a time-dependent increase in Ephrin-B2 mRNA expression in the patches. This finding suggests that the mean number of Ephrin-B2 positive cells increases within the patch in time-dependent manner. Ephrin-B2 and its receptor Eph-B4 are differentially expressed in arteries and veins, with Ephrin-B2 an arterial marker and Eph-B4 a venous marker [13], [14]. The Ephrins and the Ephs are the ligands and receptors, respectively, of the largest of the 14 subfamilies of the receptor tyrosine kinase families [15]. In the developing cardiovascular system, ephrin and Eph molecules control the angiogenic remodeling of blood vessels and lymphatic vessels and play essential roles in endothelial cells as well as in supporting pericytes and vascular smooth muscle cells [16]. However, not only are Ephrin-B2 and Eph-B4 expressed during embryonic development and determination of the vasculature, but Ephrin-B2 and Eph-B4 remain persistently expressed in adult vessels. We have previously shown that Eph-B4 is active in adult veins and regulates venous remodeling [17].

During vein graft adaptation to the arterial circulation, veins lose their expression of Eph-B4, but they also fail to express an arterial identity and do not increase expression of Ephrin-B2 or members of the delta-notch pathway [11], [18]. However, we show that pericardial patches, unlike vein grafts, are capable of gaining arterial identity, suggesting that pericardial patches can adapt to the arterial circulation. Interestingly, arterial identity appears to be expressed by cells infiltrating into the patch, whereas vein graft adaptation is characterized by intimal hyperplasia intrinsic to the vein graft, with smooth muscle cell proliferation [11].

### EPC play a role in patch remodeling

EPC play a critical role in vascular remodeling and adaptation. EPC elicit neovascularization in response to ischemia, and repair injured endothelium [19]._ENREF_15 Accordingly, autologous EPC therapy has emerged as a promising treatment option for critical limb ischemia secondary to its ability to promote neovascularization and remodeling of pre-existent vessels [20], [21], [22]. EPC are characterized by their unique surface marker expression, and although many markers have been studied and described, CD34 and VEGFR2 expression continue to be a recognized identification method [19], [23]. We used the marker CD34 to identify progenitor cells within the implanted patch, and expression of CD34 increased in a time-dependent manner from day-1 through day-7, and continued to be present in the neointima at day 30. Since the definition of EPC is not completely agreed upon, with lack of consensus as to the definitive definition and markers [24], [25], it is clear that our identification of CD34-positive cells may not definitively identify them as classic EPC. However, colocalization of CD34-positive cells with Ephrin-B2 as well as VEGFR2 (**Figure** **3**) suggests that these cells infiltrating the patch are arterial progenitor cells, although CD34 is present on surface endothelial cells, preventing our ability to distinguish EPC from endothelial cells (**Figures** **4** **,** **5**). Nevertheless, finding arterial progenitor cells infiltrating into the patch is consistent with the data that have found that coronary stent implantation may induce mobilization and differentiation of bone marrow cells into endothelial or smooth muscle cells [26]. EPC promote vascular graft patency, and circulating EPC play an important role in endothelial protection and integrity [27], [28]. Current clinical trials are attempting to exploit these characteristics by designing coated stents that attract EPC in situ, with the belief that stents that are capable of achieving this will have improved adaptation and be better equipped to heal endothelium [29], [30]. Application of these findings to the pericardial patch may help direct more EPC to the patch and aid in transfer of endothelial cells.

### Subcutaneous patches fail to attract EPC

Examination of the subcutaneously implanted patches did not reveal the presence of either CD34-positive or Ephrin-B2-positive cells, thus failing to show arterial adaptation. As compared to the arteriotomy patches, subcutaneously patches lacked exposure to arterial shear flow as well as EPC exposure from within the circulating blood pool. A confounding factor is the reduced surgical trauma and associated lesser inflammatory response of a subcutaneous implantation compared to an abdominal laparotomy, since EPC are mobilized from the bone marrow into the peripheral circulation in response to surgical trauma [26], [31].

Macrophages are known mediators of inflammation and their presence within surgical sites is well established. As expected, a large number of inflammatory cells were identified in the neighboring tissues surrounding the patch, with many cells infiltrating into the subcutaneously placed patch. We observed some macrophages infiltrating into the arterial patches, although it appeared that fewer macrophages infiltrated into the arterial patches compared to the large numbers that infiltrated into the subcutaneously placed patches.

One difference that might account for these results is the effect of the patch environment on macrophage recruitment. The extracellular matrix adjacent to the subcutaneous patch may facilitate macrophage migration as compared to the environment of the arterial patches. Arterial flow and/or pressure may exert a protective effect on the angioplasty patches that alters the inflammatory response, although the outside surface of the arterial patch is still exposed to the retroperitoneal tissues. This suggests that macrophages may need a specific signal to infiltrate the patch, or that the blood flow and/or pressure inside the vessel prevents a chemoattractive signal gradient from being established. An alternative consideration is that the early time course in this study may prevent observation of a later macrophage influx. Since our model only describes the course of healing until 30 days after implantation, longer time data points are needed for a full description of patch healing.

### Bovine and porcine patches develop neointima

Although porcine patches show earlier luminal cells compared to bovine patches (**Figure** **4**), both patches develop a similar amount of neointima on their luminal surface by 30 days (**Figure** **5**). As such, the significance of the earlier endothelialization of the porcine patches is not clear, as it may simply reflect differences in timing between bovine and porcine patch healing. Examination of bovine patches processed without gluteraldehyde could potentially examine this issue, but these patches are not currently available for surgical implantation in the vascular system. However, since both patches develop luminal neointima by 30 days, we believe that the similarities between the bovine and porcine pericardial patches, despite potential differences in their manufacturing processes, will lead to similar clinical outcomes when used in human patients. Since both patches support endothelialization and development of luminal neointima, this mode of healing predicts favorable long-term outcome, with good patency rates and minimal rates of thrombosis, infection, or pseudoaneurysm formation [3], [32].

In summary, the results from our surgical angioplasty model show that the pericardial patch is capable of supporting luminal endothelialization, promoting vessel remodeling and arterial adaptation. Ephrin-B2, CD34 and VEGFR2 positive cells were successfully identified within the implanted patch and may represent arterial or EPC. Increased Ephrin-B2 expression in the healing artery is distinctly different that the loss of Ephrin-B2/Eph-B4 expression characteristic of vein graft adaptation, suggesting that arterial and venous remodeling are fundamentally different. Arterial remodeling of pericardial patches may represent a paradigm of healing of scaffolds used for tissue engineering.

## References

[1] Bond R, Rerkasem K, AbuRahma AF, Naylor AR, Rothwell PM (2004). Patch angioplasty versus primary closure for carotid endarterectomy.. Cochrane Database Syst Rev.

[2] Bond R, Rerkasem K, Naylor AR, Aburahma AF, Rothwell PM (2004). Systematic review of randomized controlled trials of patch angioplasty versus primary closure and different types of patch materials during carotid endarterectomy.. J Vasc Surg.

[3] Muto A, Nishibe T, Dardik H, Dardik A (2009). Patches for carotid artery endarterectomy: current materials and prospects.. J Vasc Surg.

[4] Kim GE, Kwon TW, Cho YP, Kim DK, Kim HS (2001). Carotid endarterectomy with bovine patch angioplasty: a preliminary report.. Cardiovasc Surg.

[5] Matsagas MI, Bali C, Arnaoutoglou E, Papakostas JC, Nassis C (2006). Carotid endarterectomy with bovine pericardium patch angioplasty: mid-term results.. Ann Vasc Surg.

[6] Dardik H, Miller N, Dardik A, Ibrahim I, Sussman B (1988). A decade of experience with the glutaraldehyde-tanned human umbilical cord vein graft for revascularization of the lower limb.. J Vasc Surg.

[7] Dardik A, Dardik H (2011). Umbilical Vein Grafts for Lower Limb Revascularization; Bhattacharya N, Stubblefield P, editors. Regenerative medicine using pregnancy-specific biological substances.. London: Springer.

[8] Shore DF, Gabbay S, Yellin EL, Frater RW (1983). Degenerative changes in glutaraldehyde preserved pericardium used for the experimental replacement of anterior chordae of mitral valve.. J Cardiovasc Surg (Torino).

[9] Umashankar PR, Arun T, Kumari TV (2011). Short duration gluteraldehyde cross linking of decellularized bovine pericardium improves biological response.. J Biomed Mater Res A.

[10] Muto A, Model L, Ziegler K, Eghbalieh SD, Dardik A (2010). Mechanisms of vein graft adaptation to the arterial circulation: insights into the neointimal algorithm and management strategies.. Circ J.

[11] Kudo FA, Muto A, Maloney SP, Pimiento JM, Bergaya S (2007). Venous identity is lost but arterial identity is not gained during vein graft adaptation.. Arterioscler Thromb Vasc Biol.

[12] Braun J, Hoffmann SC, Feldner A, Ludwig T, Henning R (2011). Endothelial cell ephrinB2-dependent activation of monocytes in arteriosclerosis.. Arterioscler Thromb Vasc Biol.

[13] Gale NW, Holland SJ, Valenzuela DM, Flenniken A, Pan L (1996). Eph receptors and ligands comprise two major specificity subclasses and are reciprocally compartmentalized during embryogenesis.. Neuron.

[14] Adams RH, Wilkinson GA, Weiss C, Diella F, Gale NW (1999). Roles of ephrinB ligands and EphB receptors in cardiovascular development: demarcation of arterial/venous domains, vascular morphogenesis, and sprouting angiogenesis.. Genes Dev.

[15] Swift MR, Weinstein BM (2009). Arterial-venous specification during development.. Circ Res.

[16] Kuijper S, Turner CJ, Adams RH (2007). Regulation of angiogenesis by Eph-ephrin interactions.. Trends Cardiovasc Med.

[17] Muto A, Yi T, Harrison KD, Davalos A, Fancher TT (2011). Eph-B4 prevents venous adaptive remodeling in the adult arterial environment.. J Exp Med.

[18] Kondo Y, Muto A, Kudo FA, Model L, Eghbalieh S (2011). Age-related Notch-4 quiescence is associated with altered wall remodeling during vein graft adaptation.. J Surg Res.

[19] Hristov M, Weber C (2008). Endothelial progenitor cells in vascular repair and remodeling.. Pharmacol Res.

[20] Asahara T, Murohara T, Sullivan A, Silver M, van der Zee R (1997). Isolation of putative progenitor endothelial cells for angiogenesis.. Science.

[21] Tateishi-Yuyama E, Matsubara H, Murohara T, Ikeda U, Shintani S (2002). Therapeutic angiogenesis for patients with limb ischaemia by autologous transplantation of bone-marrow cells: a pilot study and a randomised controlled trial.. Lancet.

[22] Burt RK, Testori A, Oyama Y, Rodriguez HE, Yaung K Autologous peripheral blood CD133+ cell implantation for limb salvage in patients with critical limb ischemia.. Bone Marrow Transplant.

[23] Hristov M, Weber C (2004). Endothelial progenitor cells: characterization, pathophysiology, and possible clinical relevance.. J Cell Mol Med.

[24] Eggermann J, Kliche S, Jarmy G, Hoffmann K, Mayr-Beyrle U (2003). Endothelial progenitor cell culture and differentiation in vitro: a methodological comparison using human umbilical cord blood.. Cardiovasc Res.

[25] Timmermans F, Plum J, Yoder MC, Ingram DA, Vandekerckhove B (2009). Endothelial progenitor cells: identity defined?. J Cell Mol Med.

[26] Inoue T, Sata M, Hikichi Y, Sohma R, Fukuda D (2007). Mobilization of CD34-positive bone marrow-derived cells after coronary stent implantation: impact on restenosis.. Circulation.

[27] Magri D, Fancher TT, Fitzgerald TN, Muto A, Dardik A (2007). Endothelial progenitor cells: a primer for vascular surgeons.. Vascular.

[28] Sen S, McDonald SP, Coates PT, Bonder CS (2011). Endothelial progenitor cells: novel biomarker and promising cell therapy for cardiovascular disease.. Clin Sci (Lond).

[29] Kipshidze N, Dangas G, Tsapenko M, Moses J, Leon MB (2004). Role of the endothelium in modulating neointimal formation: vasculoprotective approaches to attenuate restenosis after percutaneous coronary interventions.. J Am Coll Cardiol.

[30] Siddique A, Shantsila E, Lip GY, Varma C (2010). Endothelial progenitor cells: what use for the cardiologist?. J Angiogenes Res.

[31] Wei HJ, Jiang RC, Liu L, Zhang JN (2010). Circulating endothelial progenitor cells in traumatic brain injury: an emerging therapeutic target?. Chin J Traumatol.

[32] Li X, Guo Y, Ziegler KR, Model LS, Eghbalieh SD (2011). Current Usage and Future Directions for the Bovine Pericardial Patch.. Ann Vasc Surg.

